# Successful conservative treatment of multiorgan infiltrating placenta percreta by uterine embolization and followup with serial magnetic resonance. A case report

**DOI:** 10.1016/j.ijscr.2019.04.012

**Published:** 2019-04-16

**Authors:** Ebtehaj Alshammary, Mohamed Fawaz Elmuzaini

**Affiliations:** Department of Obstetrics and Gynaecology, King Abdulaziz Medical City, King Saud bin Abdulaziz University for Health Sciences, Ministry of National Guard Health Affairs, Riyadh, Saudi Arabia

**Keywords:** Case report, Placenta percreta, Adherent placenta, Abnormal placentation

## Abstract

**Introduction:**

Conservative management was recently adopted as an option with an aim to reduce surgical complication and improve maternal outcome. We present a case of placenta percreta that invaded the urinary bladder, sigmoid colon, rectum, and vagina of a patient treated with conservative management successfully.

The work has been reported in line with the SCARE criteria (Agha et al., 2018 [1]).

**Case presentation:**

A 32-year-old woman, gravida 4, para 3. She had previously had 3 caesarean sections. At 36 week of gestation patient referred to our centre with a diagnosis of placenta praevia with a high suspicion of placenta percreta. Ultrasound and MRI repeated and confirmed the diagnosis with invasion to the rectum/sigmoid colon, urinary bladder, and upper vagina. Patient treated successfully by uterine embolization and follow up without any complication and we performed serial MRI to evaluate the course of conservative management.

**Discussion:**

Placenta percreta is a serious condition with high maternal morbidity and mortality rate. Cesarean hysterectomy is the standard treatment for now. However, conservative management was recently adopted as an option. Many case reports were published with placenta percreta that invaded the urinary bladder and treated conservatively but this is a placenta percreta that invaded the urinary bladder, sigmoid colon, rectum, and vagina. We opted conservative management and performed serial MRI to evaluate the course of conservative management.

**Conclusion:**

We believe that this case is the first case of placenta percreta that invaded the urinary bladder, sigmoid colon, rectum, and vagina. That treated successfully by conservative management and publishing this case will improve the clinical information about the course of the conservative management of placenta percreta.

## Highlights

• Morbidly adherent placenta presents sever maternal complications including death.
• This report presents a case of placenta percreta treated with conservative management without complications.
• Serial MRI was performed to evaluate the course of conservative management.

## 1 Background and importance

Morbidly adherent placenta is a rare condition [2]. Rising caesarean section rates have resulted in an increase in the incidence of placenta accreta/increta/percreta that presents a risk of massive intraoperative haemorrhage.

Maternal complications have been reported, including adjacent organ injuries, excessive blood loss with transfusion of blood products, infection, disseminated intravascular coagulation, and even death [3]. To decrease maternal morbidity in such cases, conservative management was recently adopted as an option [4].

This report presents a case of placenta percreta that invaded the urinary bladder, sigmoid colon, rectum, and vagina of a patient treated with conservative management without complications. Serial MRI was performed to evaluate the course of conservative management.

## 2 Clinical presentation

**Fig. 1:**
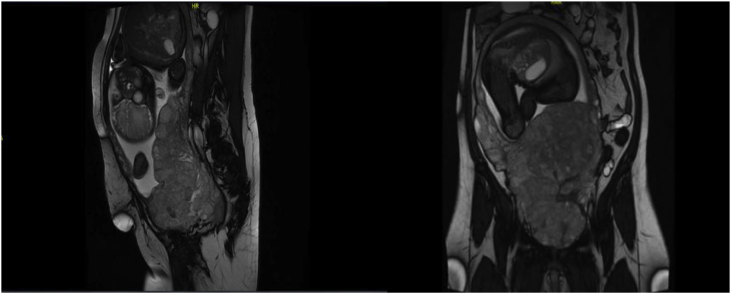
Magnetic resonance imaging showing placenta percreta with invasion of the urinary bladder fundus. There was also suspicion of invasion to the anterior wall of the rectum and lower sigmoid colon associated with congested pelvic vessels.

A 32-year-old woman presented with gravida 4, para 3, and 2 living children. She had previously had 3 caesarean sections. She was 36 weeks pregnant as confirmed by an ultrasound at 10 weeks. She was referred to our centre (Tertiary Hospital) with a diagnosis of placenta praevia with a high suspicion of placenta percreta. Ultrasound and MRI repeated at our centre confirmed the diagnosis of placenta percreta with invasion to the rectum/sigmoid colon, urinary bladder, and upper vagina (Fig. 1). The patient’s vital signs were stable and she had no vaginal bleeding, per rectum or haematuria.

## 3 Past obstetric history

The patient’s first pregnancy was complicated by placental abruption at 23 weeks with stillbirth. She was admitted to the intensive care unit due to decreased oxygen saturation and diagnosed with cardiomyopathy, but her last echocardiogram was normal. Her second and third pregnancies were straightforward caesarean sections without complications.

The patient was counselled regarding all treatment options [4,5], including conservative management. She was informed that it was newly adapted with no clear guidelines. The surgical plan and all steps were explained, and she agreed with conservative management.

Consent was obtained for high-risk treatment including bilateral tubal ligation to prevent any complications that may occur during future pregnancies.

Before surgery, the patient was cleared by a cardiologist and anaesthesiologist. Urology, surgery, and neonatology were consulted in case of possible interventions during surgery.

The blood bank prepared 6 units PRBC, 6 units of fresh frozen plasma, and 6 units of cryoprecipitate as severe bleeding was anticipated. The patient received dexamethasone to improve foetal lung maturity and 4 units PRBCs before surgery to treat her anaemia. She was taken to the surgical suite electively under general anaesthesia. The operation started with cystoscopy and bilateral ureteric catheter insertion by the urology team.

The obstetrics team (including senior consultant) made a vertical midline skin incision. The placenta was observed anteriorly infiltrating the bladder wall. Posteriorly it was not possible to reach the lower part of the pouch of Douglas as it was obscured, giving the impression of bowel invasion.

A baby boy weighing 2.420 kg with an Apgar score 7 and 9 at 1 and five minutes was delivered through an upper segment vertical incision via breech extraction. The placenta was found mainly posteriorly starting from near the fundus and descending to cover the cervix and then traversing up to the anterior uterine wall with bladder invasion.

No attempt was made to separate the placenta, which was left in situ. The cord was ligated proximal to the placental cord insertion. A tubal ligation was performed. The patient’s estimated blood loss was 500 ml, mainly from the uterine incision. Then patient was transferred to the intensive care unit in stable condition for very close observation for bleeding. There was considerable haematuria.

On postoperative day 1, the patient underwent uterine artery embolisation (UAE) that was only partially successful due to significant vascularity. A second UAE was performed on postoperative day 11.

The patient’s Foley catheter and ureteric catheters were removed on postoperative day 2 (after her haematuria abated) without complications. On postoperative day 5, she was discharged from the ICU to the ward in stable condition. Enoxaparin prophylaxis was initiated on postoperative day 7.

**Fig. 2:**
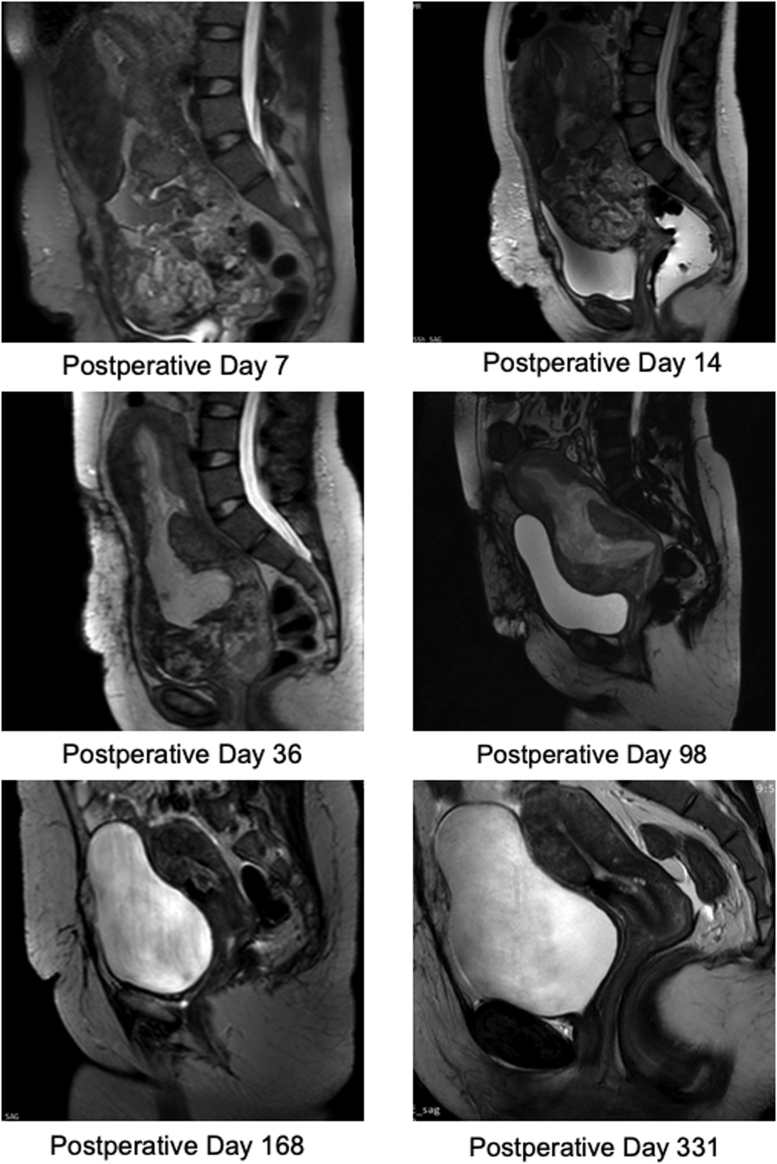
Serial magnetic resonance imaging (MRI) of conservative management of placenta percreta.

The patient remained stable. No PV, PR, or haematuria noticed on IV antibiotics + enoxaparin. She was discharged on postoperative day 19 on oral antibiotics. She had regular follow-ups with serial US and MRI for 10 months as shown in Fig. 2. During her outpatient clinic follow-up, she experienced brownish vaginal discharge with the passage of tiny pieces of tissue. She resumed her menstrual cycle 5 months after surgery.

## 4 Discussion

To the best of our knowledge, this is the first case report to demonstrate the conservative management of placenta percreta that invaded rectum/sigmoid colon, urinary bladder, and upper vagina. There were no previous reports in the literature regarding rectum invasion with term pregnancy treated conservatively. Dr. Long-Chien Lee presented a case report regarding placenta percreta with rectum invasion at 17 weeks of gestation [6].

The clinical course of the conservative management of placenta percreta remains poorly understood. A search found only 2 cases with serial MRI, both with bladder invasion only [2,7]. Therefore, it is important to observe changes to the placenta postoperatively.

Uterine artery embolisation is a well-established method employed to provide adjuvant control of intraoperative blood loss. It can also be used prophylactically to prevent delayed postpartum haemorrhage [8]. The patient in this report underwent uterine artery embolisation twice postoperatively due to high vascularity. In following the progress with serial MRI, the first improvement was noticed in the MRI on day 14 It showed no further evidence of colonic or rectal involvement, and there was a reduction in the entire placenta from 13 × 10 cm (on day 7) to 10.1 × 8.4 cm (on day 14). The first significant change in bladder invasion was noticed on day 98 and indicated placenta increta. No extension beyond the serosa was observed. A repeat MRI on postoperative day 168 demonstrated that the urinary bladder, rectum, sigmoid colon, and both ovaries appeared intact. An MRI showed that there was no placental tissue or fluid inside the endometrium. There was a clear fat plain between the uterus and the urinary bladder. The placenta had disappeared completely by postoperative day 168 (Fig. 2).

## Conflicts of interest

No potential conflict of interest relevant to this article was reported.

## Sources of funding

The authors of this study declare no sources of funding for this study.

## Ethical approval

Ethics approval was not necessary for this study and manuscript due to the type of study design (Case Report). All patient data and photographs are de-identified.

## Consent

Written informed consent was obtained from the patient for publication of this case report and accompanying images. A copy of the written consent is available for review by the Editor-in-Chief of this journal on request.

## Author contribution

Ebtehaj Alshammary: Paper Conception, Data Acquisition, Data Interpret and writing of the manuscript.

Mohamed Fawaz ِElmuzaini: supervised the work, give final approval of the version to be submitted.

## Registration of research studies

N/A.

## Guarantor

Ebtehaj Alshammary.

Mohamed Fawaz ِElmuzaini.

## Provenance and peer review

Not commissioned, externally peer-reviewed.
